# Fortifying Horticultural Crops with Essential Amino Acids: A Review

**DOI:** 10.3390/ijms18061306

**Published:** 2017-06-19

**Authors:** Guoping Wang, Mengyun Xu, Wenyi Wang, Gad Galili

**Affiliations:** 1 College of Horticulture, South China Agricultural University, Guangzhou 510642, China; gpwang@scau.edu.cn (G.W.); 11116047@zju.edu.cn (M.X.); 2 Department of Plant Science, Weizmann Institute of Science, Rehovot 76100, Israel

**Keywords:** essential amino acid, horticultural crops, nutritional quality, lysine, metabolic engineering

## Abstract

To feed the world’s growing population, increasing the yield of crops is not the only important factor, improving crop quality is also important, and it presents a significant challenge. Among the important crops, horticultural crops (particularly fruits and vegetables) provide numerous health compounds, such as vitamins, antioxidants, and amino acids. Essential amino acids are those that cannot be produced by the organism and, therefore, must be obtained from diet, particularly from meat, eggs, and milk, as well as a variety of plants. Extensive efforts have been devoted to increasing the levels of essential amino acids in plants. Yet, these efforts have been met with very little success due to the limited genetic resources for plant breeding and because high essential amino acid content is generally accompanied by limited plant growth. With a deep understanding of the biosynthetic pathways of essential amino acids and their interactions with the regulatory networks in plants, it should be possible to use genetic engineering to improve the essential amino acid content of horticultural plants, rendering these plants more nutritionally favorable crops. In the present report, we describe the recent advances in the enhancement of essential amino acids in horticultural plants and possible future directions towards their bio-fortification.

## 1. Introduction

Food quality is often defined as “everything a consumer would find desirable in a food product” [1]. In developing countries, people are prone to nutritional deficiency because plants account for the majority of their food. Therefore, improving the nutritional status of plants will help meet the dietary and micronutrient requirements of people in these countries. Conversely, in developed countries, consumers with higher income place a greater emphasis on healthy foods, such as fruits and vegetables, which provide various health compounds, such as vitamins and antioxidants, etc. Hence, improvement of the nutritional quality of horticultural crops is a relevant goal for overall sustainability.

Essential amino acids are those that are not produced in humans and farm animals; thus, they must be obtained from external sources, which are generally plants. There are nine essential amino acids, lysine (Lys), methionine (Met),threonine (Thr), phenylalanine (Phe), tryptophan (Trp), valine (Val), isoleucine (Ile), leucine (Leu), and histidine (His) [2]. The level of four of these amino acids, Lys, Met, Thr, and Trp, are the most limiting essential amino acids in plants, i.e., they are present in low quantities compared to the levels required for the optimum growth of humans and other animals [3]. Large populations in developing countries, whose diets are largely plant-derived, have insufficient levels of these essential amino acids, which can lead to major, devastating diseases. Therefore, to prevent such diseases, enriching the essential amino acid content of crops and horticultural plants (particularly fruits and vegetables), which are the major sources of human food and livestock feed in these countries is essential.

The knowledge obtained from genetic engineering research has been successfully applied to increase the content of some essential amino acids in crop plants. However, their application in horticultural crops is extremely limited. In the present report, we describe some of the approaches used to increase essential amino acid levels in horticultural crops, and evaluate the efficacy of these efforts towards the bio-fortification of horticultural plants.

## 2. Metabolism of Essential Amino Acids in Plants

**Figure 1:**
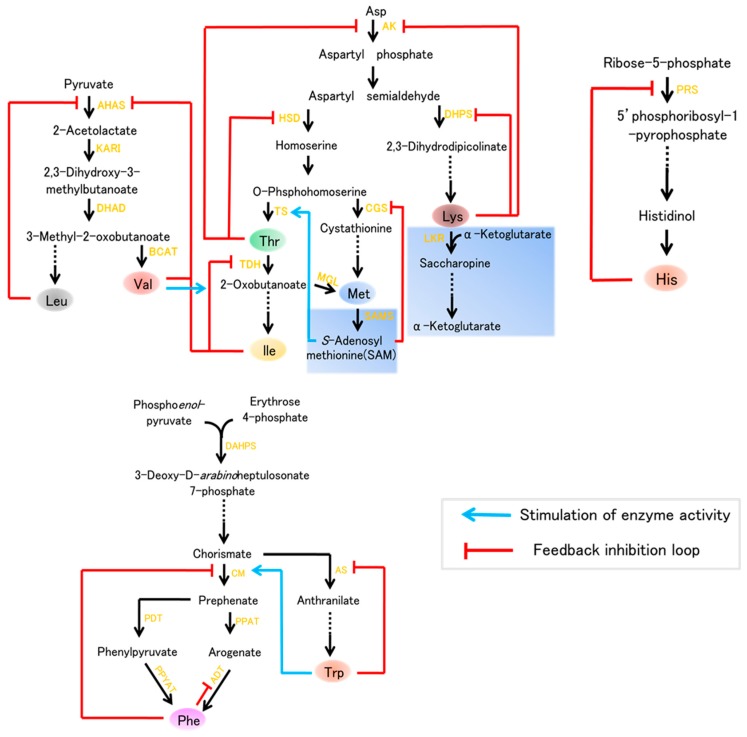
Nine essential amino acids biosynthesis leading to lysine (Lys), methionine (Met),threonine (Thr), phenylalanine (Phe), tryptophan (Trp), valine (Val), isoleucine (Ile), leucine (Leu), and histidine (His) in plants. Enzymes are indicated in yellow text. The catabolism indicated by the light blue boxes. Abbreviations: AK, Asp kinase; HSD, homoserine dehydrogenase; DHDPS, dihydrodipicolinate synthase; LKR, lysine ketoglutaric acid reductase; TS, Thr synthase; CGS, cystathionine γ-synthase; TDH, Thr dehydratase; PRS, ribose-phosphate diphosphokinase; MGL, Met γ-synthase; SAMS, S-adenosylmethionine synthase; AHAS, acetohydroxyacid synthase; KARI, ketol acid reductoisomerase; DHAD, dihydroxyacid dehydratase; BCAT, branched-chain aminotransferase; DAHPS, 3-deoxy-d-*arabino*heptulosonate 7-phosphate synthase; CM, chorismate mutase; AS, anthranilate synthase; PDT, prephenate dehydratase; PPAT, prephenate aminotransferase; PPYAT, phenylpyruvate aminotransferase; ADT, arogenate dehydratase.

The Aspartate (Asp) family of amino acids and the biosynthetic pathways for Lys, Thr, Met, and Ile in plants are shown in Figure 1. Lysine is considered to be the most limiting essential amino acid in cereals and legumes, i.e., it is present in the smallest quantity [4]. Thus, extensive efforts have been made to improve the Lys content in plants, especially in seeds, which are the major sources of human food and livestock feed in developing countries. The pathway of lysine is primarily regulated by two key enzymes, namely aspartate kinase (AK), which is the first enzyme of Asp family pathway, and the feedback-insensitive dihydrodipicolinate synthase (DHPS), which is the first enzyme specific for lysine synthesis (Figure 1). However, lysine is efficiently degraded by its catabolism in to the tricarboxylic (TCA) cycle, a pathway initiated by the bi-functional enzyme lysine-ketoglutarate reductase (LKR)/saccharopine dehydrogenase (SDH) (Figure 1). Our understanding of lysine metabolism in plants dates to the 1960s, since the discovery of the maize high-lysine mutant *opaque-2*(*o2*) which contains a low content of lysine seed storage proteins (zeins) and, consequently, increased lysine [5]. With a deep understanding of biosynthesis of lysine, a number of studies aiming to improve the content concentrated on expressing feedback-insensitive DHPS or preventing lysine degrade into the TCA cycle.

Thr, Met, and Ile, three additional essential amino acids, which are generated via another branch of the Asp family biosynthetic pathway (Figure 1), also play critical roles in plant growth and human nutrition. In plants, aspartate kinase (AK), and homoserine dehydrogenase (HSD), two key enzymes of Asp pathway, occur as either mono- or bifunctional proteins which feedback regulated by Thr. Moreover, the level of Thr is regulated by its catabolism (Figure 1). The synthesis of Met initiates from an intermediate of the Asp pathway, *O*-phosphohomoserine. Additionally, the level of Met is also regulated by its catabolic enzymes. In particular, Met is the most limiting essential amino acid in cereal and legume crops, this limitation lead to nonspecific signs of protein deficiencies in humans, such as reduced resistance to diseases [3].

The Val biosynthetic pathway starts with pyruvate, and Leu biosynthesis starts with 3-methyl-2-oxobutanoate. There are four enzymes in Val and Leu biosynthesis: acetohydroxyacid synthase (AHAS), ketol acid reductoisomerase (KARI), dihydroxyaciddehydratase (DHAD), and branched-chain aminotransferase (BCAT). Val and Leu levels are also elevated by environmental stresses and are similarly elevated by drought stress, high light, and heat stress [6,7,8]. Two essential amino acids, Trp and Phe, are important aromatic amino acids (AAA) required for protein biosynthesis that are also the precursors of various natural products in plants, such as pigments, hormones, and alkaloids [9,10]. Trp is a precursor of alkaloids, phytoalexins, and the plant hormone auxin, whereas Phe is a common precursor of numerous phenolic compounds, such as flavonoids, condensed tannins, and phenylpropanoid/benzenoid volatiles [10,11,12]. All AAAs are derived from the conversion of phosphoenolpyruvate and erythrose 4-phosphate into chorismate via the shikimate pathway; individual postchorismate pathways then lead to the synthesis of Trp and Phe (Figure 1). These pathways are present in bacteria, fungi, and plants, but are absent in animals [13]. Thus, it is important to enhance the level of these AAAs in plants which are used as human food and livestock feed.

The research on His biosynthesis in plants is far behind studies in fungi and bacteria. The His biosynthetic pathway includes nine enzymes (Figure 1), which were identified in *Arabidopsis*, and increasing evidence has implicated the critical role of ATP-phosphorilbosyl transferase in the regulation of His biosynthesis.

Although the regulation of some essential amino acid biosynthetic pathways has been extensively studied in model plants, such as Lys and Met, those in horticultural plants are not fully understood, limiting breeding and engineering efforts to improve the levels of most essential amino acids.

## 3. Fortifying Horticultural Crops with the Essential Amino Acids Lys, Met, Thr, and Trp to Improve Their Nutritional Quality

In recent decades, traditional breeding methods and mutagenesis have been applied to enhance the essential amino acid content of crop plants, and in recent years, we have obtained a detailed understanding of the enzymes involved in essential amino acid biosynthesis, degradation, and regulation in *Arabidopsis* and other model plants. These studies have made it possible to apply genetic engineering approaches for improvements of essential amino acid levels in horticultural plants. However, increasing the levels of these essential amino acids in plants, especially in horticultural plants is still difficult because (i) the synthetic pathways of some essential amino acids, such as Lys, Leu, lle, Val, Phe, Trp, are strongly regulated by a negative feedback loop; and (ii) the targeted essential amino acids are efficiently degraded by catabolism, e.g., Lys, which is degraded in the tricarboxylic (TCA) cycle [14].

Lys levels are particularly low in crops, and extensive efforts have been made to improve Lys content in *Arabidopsis* and various crops, particularly rice and maize, leading to significantly higher levels of Lys [14,15,16,17,18,19]. Since Lys biosynthesis and catabolism are well characterized in plants, it was possible to achieve desirable results in horticultural plants. A number of the earliest studies aimed to improve lysine levels in horticultural plants concentrated on expressing the lysine feedback-intensive DHPS enzymes, such as expression in potato, soybean, and canola led to a notable increase in free lysine [20,21] (Table 1). Meanwhile, plants overexpressing bacterial *DHPS* often exhibited the typical abnormal phenotype, such as a partial loss of apical dominance, delayed flowering, and abnormal leaf morphology [15]. Furthermore, Hacham et al. crossed homozygous tobacco plants overexpressing both feedback-insensitive *DHPS* and *AtCGS*, the result showed the level of lysine is similar with those expressing only *DHPS*. Unexpectedly, the level of methionine was significantly increased in plants whose co-expression of both transgenes compared with the expression of *AtCGS* alone [22]. De nova expression of α-helical coiled-coil protein also improved lysine accumulation in tobacco seeds [23]. Lys is efficiently degraded by catabolism; therefore, another effective approach for increasing Lys levels is to prevent its degradation via the TCA cycle. However, aside from the results obtained in *Arabidopsis*, maize, and rice [14,16,17,18,19], none of these approach have been successful in horticultural plants.

Met is the most limiting essential amino acid in plants, and a number of traditional breeding methods, mutagenesis, and genetic engineering approaches have been applied to enhance Met levels. However, traditional plant breeding methods have been generally met with very limited success [3]; therefore, most current efforts are focused on using genetic engineering approaches in *Arabidopsis* and horticultural plants. Similar to lysine, most attempts to elevate content have been focused on enhancing the synthesis or reducing the catabolism of Met. Overexpression of *cystathionine γ-synthase (CGS)*, which is the first enzyme in the Met biosynthesis pathway (Figure 1), led to a 6.5-, 12.8-, and 32.7-fold elevation in soluble Met content in transgenic potato, tobacco, and alfalfa leaves, respectively, revealing the regulatory role of *CGS* in Met accumulation in horticultural plants. Another effective approach for increasing Met content in horticultural plants involves the expression of sulfur-rich proteins, such as 2S albumin from Brazil nut (*Bertholletia excelsa*) and sunflower (*Helianthus annuus*). Transgenic expression of Brazil nut 2S albumin gene resulted in higher levels of Met in canola, tobacco, and *Vicia* [24,25,26,27].

Similar to Lys, Thr is synthesized through a branch of the Asp family pathway (Figure 1), and the first (Asp kinase, AK) and third (homoserine dehydrogenase, HSD) enzymes in the pathway are inhibited by Thr. Accumulating evidence has shown that AK is the major rate-limiting enzyme in Thr biosynthesis in plants. Previous studies demonstrated that mutant transgenic tobacco and alfalfa plants possessing AK or overexpressing AK showed a marked accumulation of Thr. [28,29]. Additionally, transgenic tobacco plants expressing Thr synthase (TS), the last enzyme in Thr biosynthesis, showed a five-fold accumulation of Thr [30]. Moreover, given that Thr and Met diverge from the same branch of the Asp synthesis pathway, their biosynthetic pathways compete, to some extent, for the same carbon substrate. Thus, CGS, the first unique enzyme in Met biosynthesis, likely plays an important role in Thr accumulation in horticultural plants [31].

Trp synthesis in plants is strongly regulated by feedback inhibition through the biosynthetic enzyme anthranilate synthase (AS), which catalyzes the first step in Trp biosynthesis, the conversion of chorismate to anthranilate (Figure 1). Some success has been achieved in overexpressing feedback insensitive AS to improve Trp content in *Arabidopsis* and crop plants, leading to significantly higher levels of Trp [32,33,34,35]. In horticultural plants, overexpression of *AS* is also an effective approach for enhancing Trp accumulation. Overexpression of *AS* led to a 10-fold elevation in free Trp content in transgenic tobacco and *Astragalus sinicus*, and a much greater increase (431-fold) in potato [36]. Moreover, recent studies showed that Trp levels are generally upregulated by some environmental conditions, such as light, water, and dark-induced senescence [37,38].

## 4. Conclusions

Traditional breeding has mostly failed to increase the levels of essential amino acids in plants, especially in horticultural plants, due to limited availability of genetic resources and mutants. In-depth research on the pathways of essential amino acids and their interactions with the regulatory networks in plants suggest that genetic engineering methods may be more promising. In general, three main approaches have been used to increase the levels of amino acids in plants, (i) enhance the efficiency of amino acid synthesis; (ii) prevent amino acid degradation (catabolism); and (iii) combine (i) and (ii) in the same plant. Although these approaches have been considerably successful in model and crop plants, in horticultural plants these studies are still in their infancy due to various reasons, such as the availability of efficient transformation technologies, incomplete genomic information, etc. So far, although biotechnology has been used to improve quality in crops and horticultural plants over 20 years, commercial cultivation of genetically modified (GM) crops, such as maize and cotton, has been approved in some countries, including in America, Brazil, India, and Africa [52] recently, Genome editing technology, represented by the clustered regularly interspaced short palindromic repeat(CRISPR)/Cas9 system, attain various types of genetic modification, subsequently providing a number of agricultural benefits in horticultural crops, including improvement of essential amino acids. However, as with all other GM plants, the opportunities for using such GM or genome-edited horticultural crops is dependent upon the conclusion of the public debate regarding the safety of GM food.

## Figures and Tables

**Table 1:** Increase the levels of essential amino acids lysine (Lys), methionine (Met), threonine (Thr), and tryptophan (Trp) in transgenic horticultural plants.

Amino Acid	Plant	Transgene	Tissue	Free fold Increase T/WT (Souble)	Phenotype	References
Lys	Potato	Constitutive::*DHDPS*	Leaves	2–4	ND	[20]
	Potato	Constitutive::*DHDPS*	Roots	1.2–5	ND	[20]
	Potato	Constitutive::*DHDPS*	Tubers	2–3.5	ND	[20]
	Tobacco	Constitutive::*AK*	Leaves	1.1–2	ND	[39]
	Tobacco	Constitutive::*DHDPS*	Leaves	9–11	A partial loss of apical dominance, delayed flowering and senescence, partial sterility, and abnormal leaf morphology	[15]
	Tobacco	Seed-specific::*DHDPS*	Seeds	1–2	ND	[23]
	Canola	Seed-specific::*DHDPS/AK*	Seeds	More than 100	ND	[21]
	Soybean	Seed-specific::*DHDPS/AK*	Seeds	Several hundreds	Wrinkled seed with poor germinaiton	[21]
	Soybean	*Soybean vegetative storage proteins(S-VSPs)*	Leaves	NR	ND	[40]
	Tobacco	Constitutive::*DHDPS*	Leaves	30	Mosaic green color in newly developed leaves at the tip of the apex and partial loss of apical dominance	[22]
	Tobacco	Constitutive::*DHDPS*/Constitutive::*AtCGS*	Leaves	24	ND	[22]
Thr	Tobacco	Constitutive::*AK*	Leaves	2–8	ND	[39]
	Tobacco	Constitutive::*DHDPS*	Leaves	6–8	A partial loss of apical dominance, delayed flowering and senescence, partial sterility,and abnormal leaf morphology	[15]
	Tobacco	Seed-specific::*DHDPS*	Seeds	1	ND	[23]
	Tobacco	Supress *AK*	Seeds	About 5.8	ND	[23]
	Tobacco	Seed-specific::*DHDPS/AK*	Seeds	About 3.9	ND	[23]
	Tobacco	Constitutive::*AtCGS*	Leaves	1.8	ND	[22]
	Tobacco	Constitutive::*CGS/AK*	Leaves	173	ND	[30]
Met	Tobacco	antisense *SAMS*	Leaves	433	Vein showed dark gren	[41]
	*Vicia narbonensis*	Seed-specific::*2S BN/AK*	Seeds	2	ND	[42]
	Lupin	Seed-specific::*2S SSA*	Seeds	2	Statistically significant increases in live weight gain, true protein digestibility, biological value, and net protein utilization	[26]
	Potato	Antisense *TS*	Leaves	239	Severe growth retardation, strong chlorosis, and an acute reduction in tuber yield	[31]
	Potato	Constitutive::*AtCGS*	Leaves	2–7.1	ND	[43]
	Potato	Constitutive::*AtCGS*	Root	1.2–4.1	ND	[43]
	Bean	Seed-specific::*2S BN*	Seeds	1.25	ND	[44]
	Chickpea	Seed-specific::*2S SSA*	Seeds	1.9	ND	[45]
	Alfalfa	Constitutive::*AtCGS*	Leaves	37	ND	[46]
	Tobacco	Constitutive::*DHDPS*	Leaves	2	ND	[22]
	Tobacco	Constitutive::*AtCGS*	Leaves	5	ND	[22]
	Tobacco	Constitutive::*DHDPS*/Constitutive::*AtCGS*	Leaves	10	ND	[22]
	Tobacco	Constitutive::*CGS/AK*	Leaves	39		[22]
	Potato	Constitutive::*AtCGS/zein*	Tubers	2–6	ND	[47]
	Tobacco	Constitutive::*CGS/AK*	Leaves	39	ND	[30]
	Soybean	Seed -specific::*AtCGS*	Seeds	2.1	ND	[48]
	*Azuki* bean	Seed -specific::*AtCGS*	Seeds	2.3	ND	[48]
Trp	Astragalus	Constitutive::*ASA2*	Hariy roots	1.3–5.5	ND	[49]
	Tobacoo	Constitutive::*ASA2*	Leaves	16	ND	[50]
	Tobacco	Constitutive::*CGS/AK*	Leaves	39	ND	[30]
	Soybean	Seed -specific::*OASA1D*	Seeds	50.9	ND	[51]

Abbreviations: DHDPS, feedback-insensitive dihydrodipicolinate synthase; AK, feedback-insensitive Asp kinase; AtCGS, *Arabidopsis thaliana* cystathionine γ-synthase; TS, Thr synthase; 2S SSA, 2S sunflower seed albumin; 2S BN, 2S brazil nut albumin; OASA1D, rice mutated feedback-resistant a subunit of rice anthranilate synthase; NtASA2, tobacco feedback-insensitive anthranilate synthase 2; ND, not detected.

## Abbreviations

T	Transgene
WT	Wild type
SS	Seed-specific promoter
Sup	Suppression the gene by antisense or RNAi
M	Mutant
NR	Not reported
NS	Not significant
SSP	Seed storage protein
AtCGS	*ARABIDOPSIS thaliana* cystathionine γ-synthase
AtD-CGS	Feedback insensitive form of AtCGS
EcSAT	*E. coli* serine acetyl transferase
SAMS	S-adenosyl Met synthase
MGL	Met γ-lyase
TS	Thr synthase
2S SSA	2S sunflower seed albumin
2S BN	2S brazil nut albumin
bAK	Bacterial feedback-insensitive Asp kinase
bDHDPS	Bacterial feedback-insensitive dihydrodipicolinate synthase
LKR	Lys-ketoglutarate reductase
Bip	Lys-rich binding protein
sb401	Lys-rich pollen-specific protein
RLRH	Rice Lys-rich histone proteins
OASA1D	Rice mutated feedback-resistant a subunit of rice anthranilate synthase
NtASA2	Tobacco feedback-insensitive anthranilate synthase 2
